# Transgenerational changes in pod maturation phenology and seed traits of *Glycine soja* infested by the bean bug *Riptortus pedestris*

**DOI:** 10.1371/journal.pone.0263904

**Published:** 2022-03-02

**Authors:** Shuhei Adachi-Fukunaga, Yui Nakabayashi, Makoto Tokuda

**Affiliations:** 1 The United Graduate School of Agricultural Sciences, Kagoshima University, Kagoshima, Japan; 2 Department of Biological Resource Science, Saga University, Saga, Japan; Zhejiang University, CHINA

## Abstract

Land plants have diverse defenses against herbivores. In some cases, plant response to insect herbivory may be chronological and even transgenerational. Feeding by various stink bugs, such as the bean bug *Riptortus pedestris* (Hemiptera: Alydidae), induce physiological changes in soybean, called as green stem syndrome, which are characterized by delayed senescence in stems, leaves, and pods. To investigate the plant response to the bean bug feeding in the infested generation and its offspring, we studied the effects of *R*. *pedestris* infestation on *Glycine soja*, the ancestral wild species of soybean. Field surveys revealed that the occurrence of the autumn *R*. *pedestris* generation coincided with *G*. *soja* pod maturation in both lowland and mountainous sites. Following infestation by *R*. *pedestris*, pod maturation was significantly delayed in *G*. *soja*. When *G*. *soja* seeds obtained from infested and non-infested plants were cultivated, the progeny of infested plants exhibited much earlier pod maturation and larger-sized seed production than that of control plants, indicating that *R*. *pedestris* feeding induced transgenerational changes. Because earlier seed maturity results in asynchrony with occurrence of *R*. *pedestris*, the transgenerational changes in plant phenology are considered to be an adaptive transgenerational and chronological defense for the plant against feeding by the stink bug.

## Introduction

Land plants have extensive defense systems against herbivorous insects [1–3], including not only direct defenses such as physical and chemical traits, but also indirect biotic defensive traits [4]. These defenses are either constitutive or induced in response to insect attack [5,6]. Plant response to insect herbivory may be chronological, such as in phenological escape [7–9], or even transgenerational [10,11]. Transgenerational changes include reinforcement of physical and chemical defense traits in offspring in response to insect infestation of parental generations [12–14]. These studies have been conducted mainly in model plants or crops [11], and rarely in wild plants [10]. However, the laboratory-reared plant strains or domesticated crops may not be suitable to understand the impact of transgenerational changes in natural populations because domestication is often conducted in different environments including new abiotic condition, cropping system, and interspecific interaction [15–17]. In addition, human selection strongly influences phenotypic variation of domesticated plants, which may cause changes in plant responses to insect herbivory [18] and abiotic stress [19,20]. Therefore, studies using wild plants are essential to understand the adaptive significance of transgenerational changes in plant defense.

Soybean *Glycine max* (L.) Merr. (Fabaceae), domesticated from the latter 5,000–9,000 years ago in East Asia [21–23], is one of the most important crops cultivated widely in North and South America as well as in Asian countries. In this plant, sometimes physiological damage called as green stem disorder occurs by depodding and thinning. This phenomenon represents normal, mature pods on green soybean stems, and is considered as effect of source-sink balance, that is, a relative increase in source levels by reductions in sink size [24]. A similar damage is also induced by feeding of various stink bugs or viruses, which is known as green stem syndrome or green soybean syndrome [24] and is defined by the delayed senescence of stems, leaves, and pods as well as the increase of sterile pod ratio [25,26]. Although the detailed mechanism of green stem syndrome is not yet clarified, the symptom occurs not only due to source-sink imbalance [26].

*Glycine soja* Siebold & Zucc. (Fabaceae), the wild species of soybean *G*. *max*, is an annual plant widely distributed in Japan [27,28]. In Japan, *G*. *soja* is often attacked by the bean bug *Riptortus pedestris* F. (Hemiptera: Alydidae), which is a serious soybean pest causing green stem syndrome in East Asia [29–35]. *Riptortus pedestris* utilizes various plants of Fabaceae as hosts especially after flowering. For example, the bean bug is often confirmed on *Astragalus sinicus　*L. and *Trifolium pratense* L. from spring to summer [36], and on *G*. *soja* and *G*. *max* from summer to autumn [36,37]. On the host plants, *R*. *pedestris* inserts its stylet (needle-like mouthpart) into stems, leaves, flowers and pods [35]. In this study, we focus on physiological response of *G*. *soja* to feeding damage by *R*. *pedestris* on the parental (= damaged) generation and its offspring. Because, as mentioned above, the physiological responses of soybeans against stink bugs could have been altered by the process of crop breeding, we used the wild counterpart *G*. *soja* to detect the natural reaction of plants to *R*. *pedestris* infestation. We first investigated the seasonal occurrence of the bean bug *R*. *pedestris* and pod maturation phenology of *G*. *soja* in the field to compare the chronological relationship between *R*. *pedestris* feeding and *G*. *soja* pod production. Second, we surveyed effects of feeding by *R*. *pedestris* on pod maturation phenology and seed traits of *G*. *soja*. Third, we evaluated transgenerational effects of *R*. *pedestris* infestation on the phenology and seed traits of *G*. *soja*. Based on these results, we discuss adaptive significance of transgenerational changes in pod maturation phenology and seed traits of *G*. *soja*.

## Materials and methods

### Plants and insects

Mature seeds of *G*. *soja* were collected from natural communities growing in Ogori City, Fukuoka Prefecture (33°26.33’ N, 130°33.49’ E) (Fukuoka strain) in October 2012 and Saga City, Saga Prefecture (33°23.95’ N, 130°16.30’ E) (Saga strain) in autumn 2013. A laboratory reared *R*. *pedestris* strain originally collected from a soybean crop in Koshi City, Kumamoto Prefecture in 2003 was used in the present study. The strain was continuously reared in the laboratory according to a previous study [38], in which dry soybean seeds and water (with filter paper) was supplied as food and wool yarn as oviposition substrate in plastic cages and the cages were maintained at 25 ˚C and under a 16-hour light: 8-hour dark photoperiodic condition. No permits were required for the collection of plants and insects at the study sites. Our studies did not involve any endangered or protected species.

### Seasonal occurrence of *R*. *pedestris* and field phenology of *G*. *soja*

To confirm the seasonal occurrence of *R*. *pedestris* and reproductive phenology of *G*. *soja*, periodic field investigations were conducted at two census sites, Honjo (on Saga University campus, as a lowland site; 33°14.58’ N, 130°17.40’ E; alt. 12m) and in Mitsuse (as a mountainous site; 33°23.95’ N, 130°16.30’ E; alt. approximately 400 m), Saga Prefecture, northern Kyushu, Japan. Three box traps each with four pieces of sticky sheets (FIELDCATCH, Fuji Flavor, Tokyo, Japan) were placed near naturally growing populations of *G*. *soja* in each site from April to November 2017. Commercially available synthetic aggregation pheromone lures for *R*. *pedestris* (Fuji Flavor, Tokyo, Japan) were used as attractants for the traps [39]. Then the number of *R*. *pedestris* captures was recorded at two-week intervals. Sticky sheets were replaced on every census day. The number of flowers and seed pods of *G*. *soja* as well as pod maturity (evaluated by the color of seed pods) was surveyed on each census day.

### Effects of *R*. *pedestris* infestation on pod maturation phenology and seed traits

Seeds of *G*. *soja* obtained from our field sites in Fukuoka and Saga were sown on 26th June 2015 in vinyl pots (13.5cm diameter 11cm high) containing 70% red soil and 30% humus. Seeds were cultivated in an unheated greenhouse on the Saga University Campus, and sufficiently watered every day. Greenhouse windows were covered with 1-mm mesh cloth for ventilation. Seedlings were covered with 4-mm mesh net (40 cm height and 16 cm diameter). During the flowering season, either 0 (control; n = 10), 6 (low-density treatment; n = 5 plants), or 12 (high-density treatment; n = 4 plants) female *R*. *pedestris* adults were placed on each plant for nine days. All adults and eggs were removed after nine days. When the flowers developed into green pods, 20 pods on each plant were arbitrarily covered with mesh bags (95 mm × 70 mm) to collect seeds and examine the number of seeds and the dry seed mass after pod dehiscence. Matured black pods and sterile black pods were then counted and collected at one- to three-day intervals from 15th October 2015 until all pods matured. Seed production was estimated from the number of seeds in the 20 covered pods and the total number of pods on each plant. Seeds collected from the 20 pods were individually weighed and the dry seed mass was evaluated in each pod.

### Transgenerational effects of *R*. *pedestris* infestation on pod maturation phenology and seed traits

In this experiment, only Saga strain was used, because in the parental generation of *G*. *soja*, significant effects of *R*. *pedestris* feeding were detected only in this strain (see results). *Glycine soja* seeds were sown on 20th June 2016 and cultivated as in the preceding experiments. The seedings were exposed to 0 (control; n = 3 plants) or 12 (RP treatment; n = 2 plants) adult female *R*. *pedestris* for nine days of the flowering season. After nine days, all adults and eggs were removed. Seeds were collected from respective plants in late October 2016, stored at 5 ˚C, sown on 13th June 2017, and cultivated in the greenhouse as in the preceding experiments but without exposure to *R*. *pedestris*. Two seeds were planted in each of 15 pots, and one seedling remained after recording the germination rates and days. Seeds collected from three control and two *R*. *pedestris-*infested parental plants were randomly used. When the flowers developed into green pods, 10 pods from each plant were arbitrarily covered with mesh bags (95 mm × 70 mm) to examine pod and seed traits. Matured black pods were then counted and collected weekly in October and November 2017. Seed production was estimated from the number of seeds in the 10 covered pods and the total number of pods on each plant. Seed weight was measured from seeds in the 10 covered pods.

### Statistics

Days from the sowing to seed maturity (pod maturation time) were analyzed by a generalized linear mixed model (GLMM) with a Poisson distribution. The ratio of sterile pods (pods with no mature seeds) was analyzed by a GLMM with a binomial distribution and a log link function. In the models, *R*. *pedestris* treatments were included as the fixed effect and plant individuals as the random effect. In addition, origin of seeds (Fukuoka or Saga) was included as a fixed effect in the analysis of the sterile pod ratio. The numbers of pods on plants, mature seeds on plants, and mean number of seeds per plant as well as days until germination of the next generation of *G*. *soja* were analyzed by a generalized linear model (GLM) with a Poisson distribution and a log link function. Weight of mature seeds was analyzed by GLM with a Gamma distribution and an inverse link. Days from sowing to germination (germination days) in the next generation of *G*. *soja* were analyzed by a GLM with a Poisson distribution. In GLMMs and GLMs, treatment means were compared by Tukey’s honestly significant difference (HSD) test. Germination rate of *G*. *soja* in the next generation was analyzed by Fisher’s exact probability test. All statistical analyses were performed using R ver. 3.5.1 [40].

## Results

### Seasonal occurrence of *R*. *pedestris* and field phenology of *G*. *soja*

**Fig 1:**
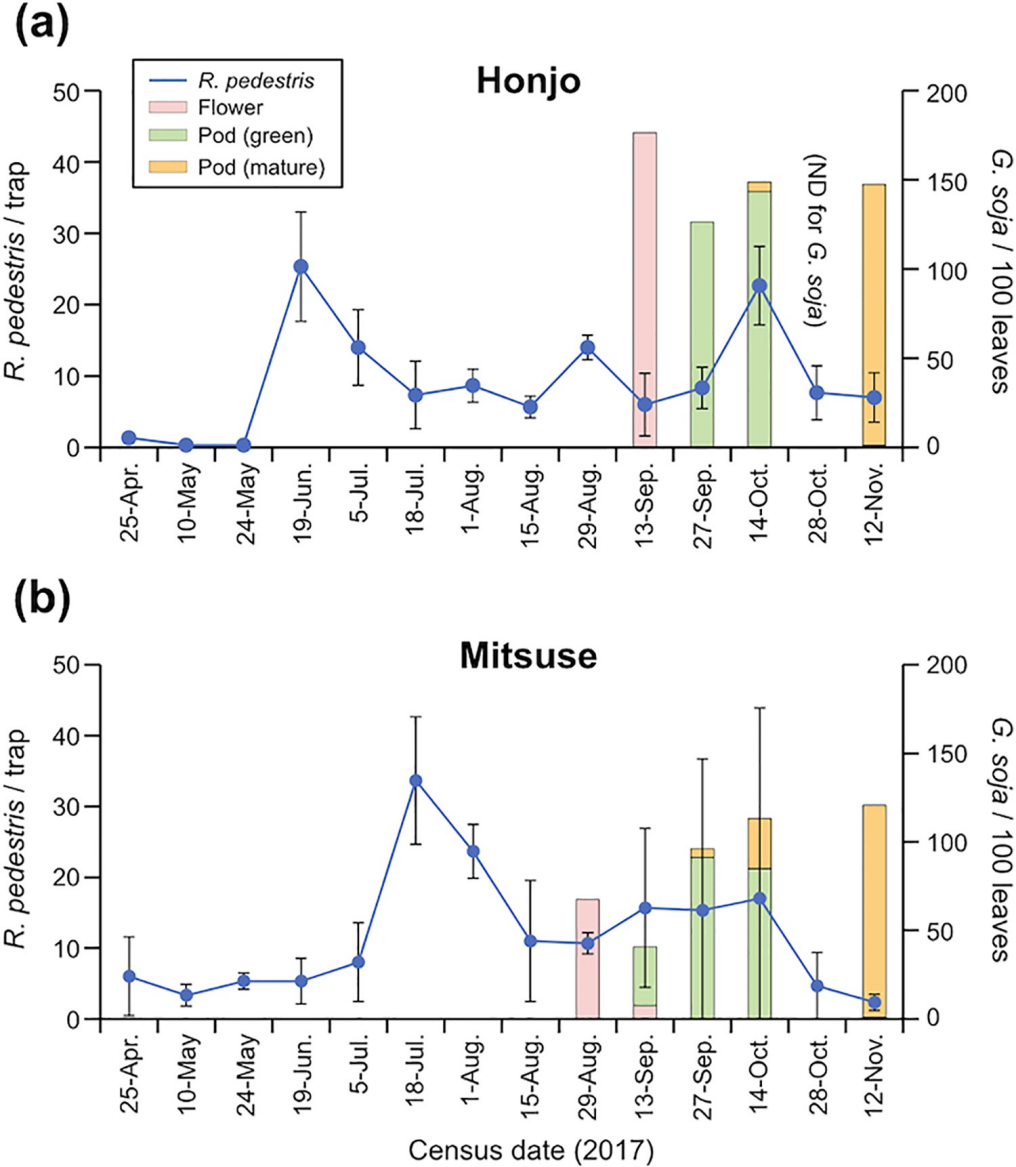
Seasonal trends (blue lines) of *Riptortus pedestris* captured by pheromone traps and pod maturation phenology of *Glycine soja* (bars) at the two census sites in Saga, northern Kyushu, Japan. Pink, green and yellow bars indicate the number of flowers, green pods and matured pods per 100 leaves, respectively.

Both in Honjo (Fig 1A) and in Mitsuse (Fig 1B), seasonal trends of *R*. *pedestris* captured by pheromone traps had a few peaks, including June, August and October in Honjo, and July and September-October in Mitsuse (Fig 1). *Glycine soja* flowers appeared in mid-September in Honjo, and in late August in Mitsuse (Fig 1). Pods began to mature in mid-October in Honjo, and late September in Mitsuse (Fig 1). The beginning of pod maturation periods of *G*. *soja* coincided with the autumn peaks of *R*. *pedestris* captures.

### Effects of *R*. *pedestris* infestation on pod maturation phenology and seed traits

**Fig 2:**
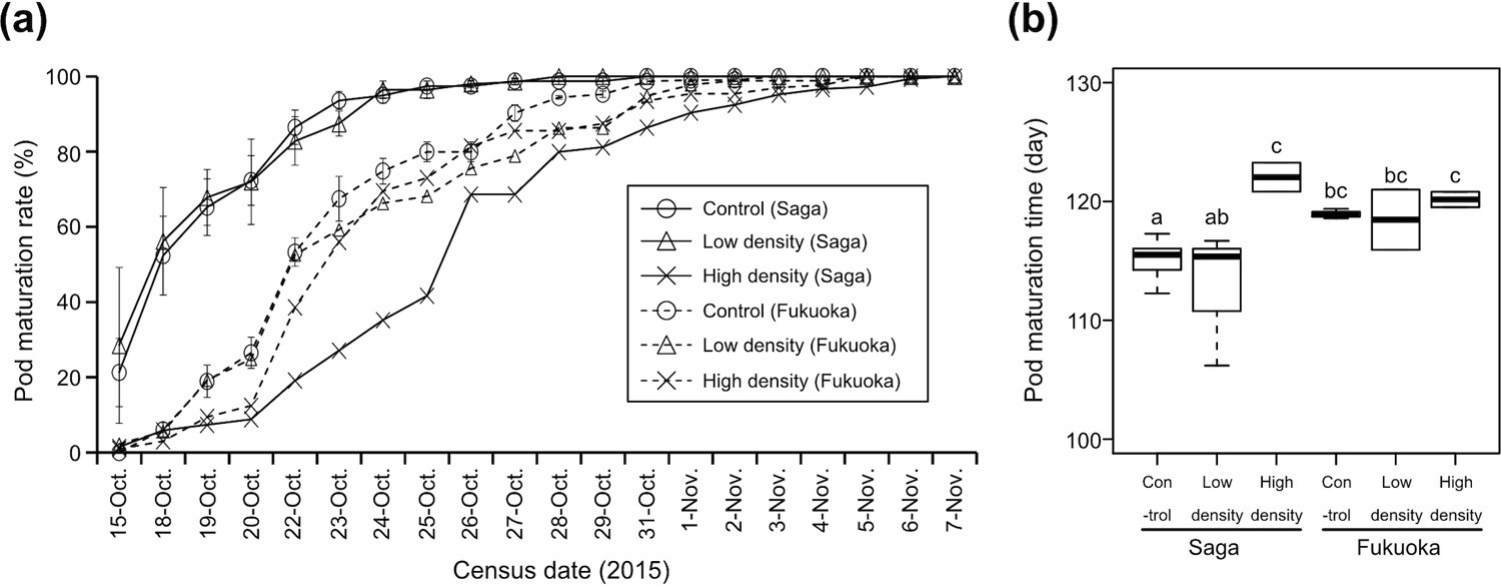
(a) Pod maturation rate and (b) pod maturation time of *Glycine soja* infested with 0 (control), 6 (low density) or 12 (high density) *Riptortus pedestris* females. In Fig 2A, the solid and dotted lines depict the experiment of Saga and Fukuoka strains, respectively. In Fig 2B, the different letters above bars indicate significant differences between treatments (Tukey’s HSD test; p < 0.05).

In Saga strain, pod maturation rate reached 50% in 18th October in control and low-density treatment, while it was 5.9% in high-density treatment (Fig 2A). All pods were mature in 31st, 28th October and 7th November in control, low-density and high-density treatments, respectively (Fig 2A). In Fukuoka strain, pod maturation rate reached 50% from 22nd to 23rd October in all treatments (Fig 2A). All pods were mature from 3rd to 5th November in all treatments (Fig 2A). Insect feeding, seed origin, and their interaction significantly affected pod maturation times (GLMM; χ^2^ = 28.65, Df = 2, p < 0.001 for *R*. *pedestris* (RP) treatment; χ^2^ = 10.91, Df = 1, p < 0.001 for origin; χ^2^ = 15.93, Df = 2, p < 0.001 for RP treatment × origin). Pod maturation time was significantly longer in plants originating from Fukuoka relative to plants originating from Saga in control (Tukey’s HSD test; p < 0.05) (Fig 2B). In Saga strain, it was significantly longer in the high-density treatment than in control and the low-density treatment (Tukey’s HSD test; p < 0.05) (Fig 2B). In contrast, no significant differences were detected between treatments in Fukuoka strain (Tukey’s HSD test; p = 0.99 between control and low-density treatments, p = 0.95 between control and high-density treatments, p = 0.99 between low-density and high-density treatments) (Fig 2B).

**Fig 3:**
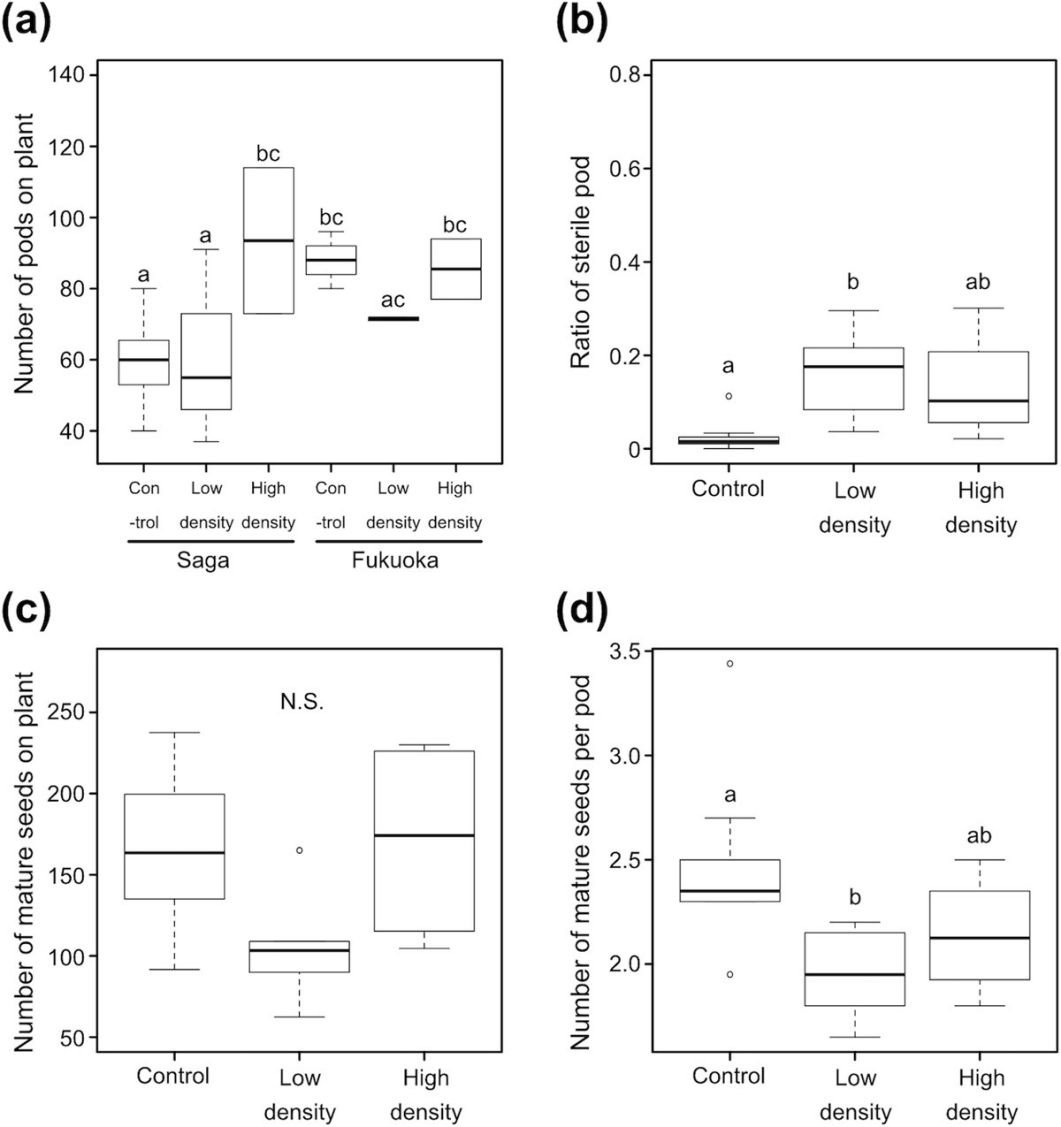
Pod and seed production in *Glycine soja* infested with 0 (control), 6 (low density) or 12 (high density) *Riptortus pedestris* females. (a) Number of pods on each plant in Saga and Fukuoka strains. (b) Ratio of sterile pods among collected pods. (c) Number of matured seeds on each plant. (d) Number of matured seeds per pod. The different letters above the error bars indicate significant differences between treatments (GLM; p < 0.05, Tukey’s HSD test; p < 0.05). N.S., not significant.

The number of pods produced by plants was significantly affected by *R*. *pedestris* infestation and plant origin (GLMM; χ^2^ = 16.92, Df = 2, p < 0.001 for RP treatment; χ^2^ = 13.11, Df = 1, p < 0.001 for origin; χ^2^ = 13.38, Df = 2, p < 0.01 for RP treatment × origin). The number of pods was significantly higher in the high-density group than in the control and low-density groups in the Saga strain of *G*. *soja*, but no significant differences were detected in the Fukuoka strain (Fig 3A). The ratio of sterile pods was significantly affected by *R*. *pedestris* infestation (GLMM; χ^2^ = 22.95, Df = 2, p < 0.0001 for RP treatment; χ^2^ = 2.77, Df = 1, p = 0.10 for origin; χ^2^ = 4.43, Df = 2, p = 0.11 for RP treatment × origin) (Fig 3B). The ratio was significantly higher in the low-density group (p < 0.001), and non-significantly increased in the high-density group (p = 0.095) relative to the control group (Tukey’s HSD test) (Fig 3B). Seed production was significantly affected by *R*. *pedestris* infestation (GLMM; χ^2^ = 6.91, Df = 2, p < 0.05 for RP treatment; χ^2^ = 1.75, Df = 1, p = 0.19 for origin; χ^2^ = 1.30, Df = 2, p = 0.52 for RP treatment × origin). Although no significant differences were detected in pairwise comparisons, seed production tended to have decreased in the low-density group relative to control (p = 0.08, Tukey’s HSD test) (Fig 3C).

The number of mature seeds per pod was significantly affected by *R*. *pedestris* infestation (GLMM; χ^2^ = 8.25, Df = 2, p < 0.05 for RP treatment; χ^2^ = 1.15, Df = 1, p = 0.28 for origin; χ^2^ = 1.32, Df = 2, p = 0.51 for RP treatment × origin). Seeds per pod significantly decreased only in the low-density group relative to control (Fig 3D). The seed origin (Fukuoka or Saga) significantly affected mean seed weight (mean ± SD = 18.7 ± 0.67 and 21.1 ± 2.13 mg in the Fukuoka and Saga strains, respectively), but the effect of RP treatment on seed weight was not significant (mean ± SD = 20.8 ± 1.81, 19.6 ± 1.99 and 19.8 ± 3.07 mg in the control, low-density and high-density treatments, respectively) (GLMM; χ^2^ = 0.89, Df = 2, p = 0.64 for RP treatment; χ^2^ = 6.89, Df = 1, p < 0.01 for origin; χ^2^ = 0.02, Df = 2, p = 0.99 for RP treatment × origin).

### Transgenerational effects of *R*. *pedestris* infestation on pod maturation phenology and seed traits

**Fig 4:**
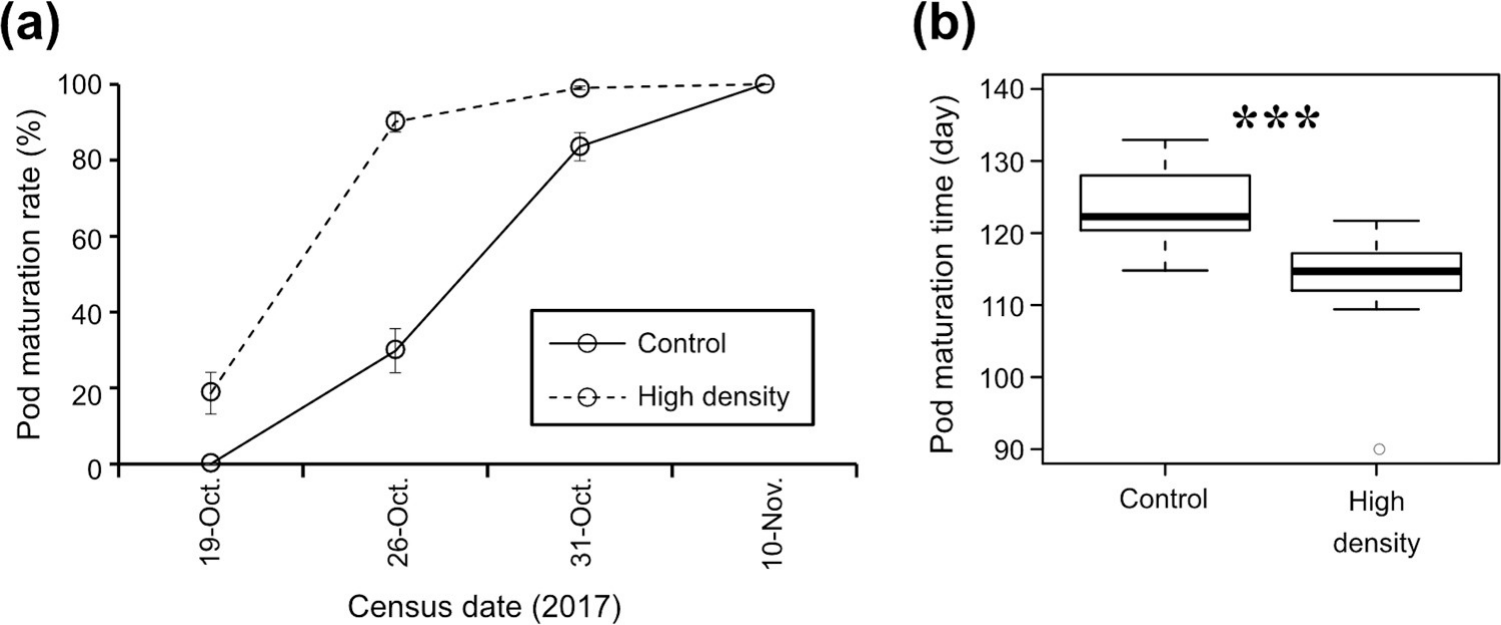
(a) Pod maturation rate and (b) pod maturation time of *Glycine soja* progeny of infested (high density) or non-infested (control) plants. Asterisks indicate significant differences between treatments (GLMM; p < 0.0001).

In the next generation of *G*. *soja*, germination rates (100% in the control and 90% in the RP treatment; n = 30 for each) (Fisher’s exact probability test; p = 0.237) and germination days (mean ± SD = 5.1 ± 1.6 and 6.0 ± 1.9 days in the control and high-density treatment, respectively) (GLM; χ^2^ = 2.08, Df = 1, p = 0.149) were not significantly different between the control and the high-density treatment progeny. In contrast to the preceding generation, pod maturation rate was around 30% in the control progeny on 26th October while it reached 90% in the high-density treatment progeny on the same day (Fig 4A). All pods were mature on 10th November in both treatment progenies (Fig 4A). Pod maturation time was significantly decreased in the high-density treatment progeny relative to the control progeny (GLMM; χ^2^ = 53.93, Df = 1, p < 0.0001) (Fig 4B).

**Fig 5:**
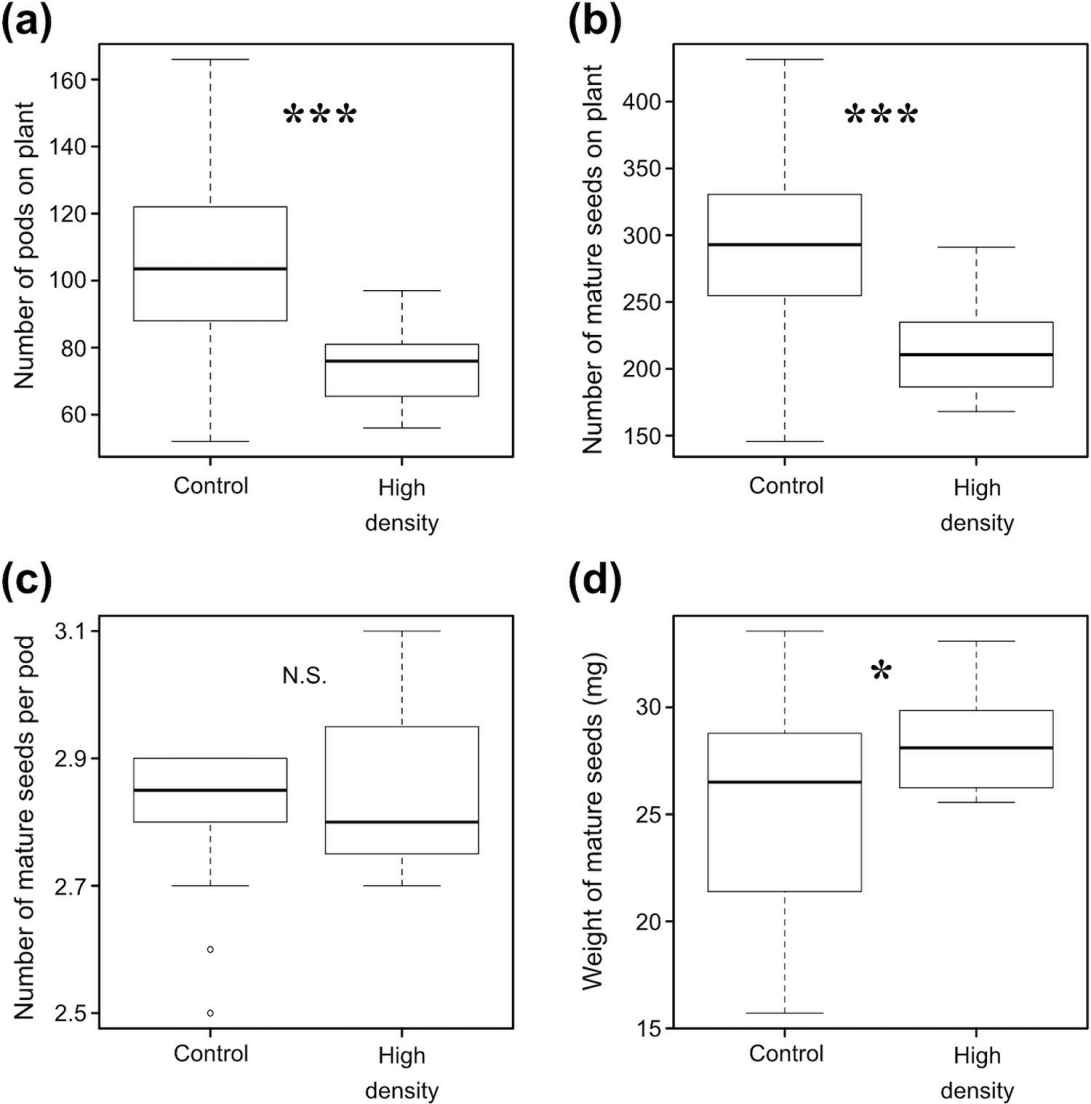
Pod and seed production in the progeny of *Riptortus pedestris* infested (high density) and non-infested (control) *Glycine soja*. (a) Number of pods on each plant. (b) Number of mature seeds on each plant. (c) Number of mature seeds per pod. (d) Mean weight of mature seeds. Asterisks indicate significant differences between treatments (GLM; *p < 0.05, ***p < 0.001; N.S., p > 0.05).

The numbers of pods and mature seeds were significantly fewer in the RP progeny than in the control progeny (GLM; χ^2^ = 75.79, Df = 1, p < 0.0001 for number of pods; χ^2^ = 18.52, Df = 1, p < 0.0001 for number of mature seeds) (Fig 5A and 5B). The ratio of sterile pods (mean ± SD) was not significantly different between RP treatment (0.011 ± 0.034) and control progeny (0.015 ± 0.020) (GLMM; χ^2^ = 1.95, Df = 1, p = 0.162). Although the number of mature seeds per pod was not significantly different between the RP treatment and control progeny (GLM; χ^2^ = 0.91, Df = 1, p = 0.341) (Fig 5C), seed weight was significantly higher in RP treatment progeny than in control progeny (GLM; χ^2^ = 4.68, Df = 1, p < 0.05) (Fig 5D).

## Discussion

In this study, we first clarified the chronological relationship between *R*. *pedestris* feeding and *G*. *soja* pod production. In Japan, *R*. *pedestris* has 2–3 annual generations [36]. Our field study confirmed three peaks of *R*. *pedestris* in Hojo, and two peaks in Mitsuse, suggesting that this species likely has three generations in the lowlands, and two generations in the mountainous areas of the Saga Prefecture. A previous study [41] demonstrated that the peak incidence of *R*. *pedestris* occurred in early September when the soybean pods began to enlarge. In addition, the number of *R*. *pedestris* in soybean field reached a peak one month before the peak incidence in water-pan traps with synthetic attractants [41]. In our study sites, the last peaks of *R*. *pedestris* captures coincided with green and mature pod stages of *G*. *soja*. These suggest that *R*. *pedestris* infestation reached a peak in flower and green pod stages of *G*. *soja* in our census field.

As previously mentioned, green stem syndrome is characterized as delayed senescence of stems, leaves, and pods as well as the increase of sterile pod ratio [25,26]. Our *R*. *pedestris* infestation treatments demonstrated the delayed senescence of pods, and the increase of sterile and mature pods. Therefore, we concluded these changes were green stem syndrome of *G*. *soja*. Interestingly, *G*. *soja* of the Saga strain was clearly more susceptible to *R*. *pedestris* feeding than that of the Fukuoka strain. Some studies demonstrated that the susceptibility of *G*. *soja* to photoperiod and temperature differs between varieties depending on the region in which it is originally grown [42,43]. Similarly, local adaptation to herbivore attacks or other factors may affect the susceptibility of the Saga and Fukuoka strains. In some *G*. *soja* traits, inconsistency was confirmed between the intensity of RP treatment and its effects. Although no significant differences were detected in the ratio of sterile pod and the number of mature seeds per pod in the high-density treatment, they were respectively increased and decreased in the low-density treatment. One possible reason for this inconsistency is an extension of the vegetative growth period in the high-density treatment due to delayed pod maturation observed only in this treatment. Unlike the natural context of the field, in this study we removed all insects and eggs from plants after nine days of exposure, which might enable the compensation of plants in the high-density group. From an adaptive point of view, green stem syndrome is disadvantageous for plants because it increases sterile pods and reduces yield. A previous study demonstrated that, in soybean seeds infested by *R*. *pedestris*, protein levels increased but lipid and carbohydrate contents and germination potential decreased [31]. The present study also detected that extension of the pod maturation period, in which insects can directly damage seeds contained in the pods, is disadvantageous for plants.

On the contrary to the parental generation, pod maturation time of the progeny of infested *G*. *soja* was significantly earlier than the control. Because germination rates and germination days were not significantly different between the control and the high-density treatment progeny, germination timing is not considered to affect this early pod maturation. The earlier reproduction can cause asynchrony between seed maturation of *G*. *soja* and emergence of the *R*. *pedestris* autumn generation. The effect of asynchrony has been intensively studied in several lepidopteran species [44–49] as well as in some other insects [50,51]. For example, relative fitness of the winter moth *Operophtera brumata* (Geometridae) decreased in phenological asynchrony with *Quercus robur* (Fagaceae) [49]. The early hatching of *O*. *brumata* leads to increased mortality, whereas the later hatching results in decrease of the host plant quality and thereby decreased fecundity of *O*. *brumata*. In another study, a phenological asynchrony induced by the delay in the larval occurrence of gypsy moth *Lymantria dispar* (Erebidae) and their feeding on mature silver birch *Betula pendula* (Betulaceae) leaves caused negative effects on the larval development rate and the female pupal weight. In addition, the larval susceptibility to exogenous nucleopolyhedrovirus infection was enhanced due to the phenological asynchrony [47]. Although the severity of phenological asynchrony varies among host plants and herbivores [48], these studies indicate that the phenological change observed in *G*. *soja* progeny is possible to reduce the damage of *R*. *pedestris*. The reproductive period is also influenced by the photoperiod and temperature [42]. For example, an increased difference between daytime and nighttime temperatures enhances the flowering of high latitude varieties of *G*. *soja* [43]. In fact, the flowering of *G*. *soja* was earlier in Mitsuse located in a mountainous area than in Honjo located in a lowland in our field investigations. Future studies are needed to clarify the effect of *R*. *pedestris* feeding under the conditions promoting flowering or maturity by other factors. The total number of pods and seeds significantly decreased in progeny of the RP treatment groups, while the mean seed weight significantly increased. In various plants, larger-sized seeds are more tolerant than small-sized ones against seed predation by herbivores [52–54]. Therefore, the increase of *G*. *soja* seed weight may also be a defensive reaction against *R*. *pedestris*.

In summary, we surveyed the seasonal occurrence of *R*. *pedestris* and pod maturation phenology of *G*. *soja* in the field and revealed that the occurrence of the *R*. *pedestris* autumn generation coincided with the flower and green pod season of *G*. *soja*; feeding by *R*. *pedestris* induced a phenological delay in pod maturation and changes in pod and seed traits in *G*. *soja*; and the progeny of *G*. *soja* infested by *R*. *pedestris* had contrasting phenological changes to those of the parental generation, including earlier pod maturation and large-sized seed production. Because *R*. *pedestris* infestation was allowed only when the parental generation of *G*. *soja* was a flowering season (i.e., before pod production), we conclude that the phenotypic changes in the progeny are transgenerational responses. In addition, these transgenerational changes are expected to avoid damage by *R*. *pedestris* and also to result in higher tolerance against seed predation in *G*. *soja*.

In general, transgenerational changes are known to occur through several steps [12,55]. In the first step, parental generation receives environmental cues, including insect herbivory and hormonal defense induction occurs. In the second step, phloem-mobile small RNA, in addition to small molecules, provide a signal that allows transfer of information from vegetative tissue to developing seeds. Finally, chromatin modifications enabling phenotypic changes, such as DNA methylation and histone acetylation, occur in offspring. A previous study demonstrated that a mirid bug *Tupiocoris notatus* possesses very high levels of cytokinins and transfers their phytohormones into a host plant *Nicotiana attenuata* to alter source/sink relationships [56]. Based on our preliminary experiments (M. Tokuda et al. unpublished), *R*. *pedestris* possesses certain amounts of phytohormones auxin and cytokinins, and the concentration of cytokinins increases in soybeans after infestation by *R*. *pedestris*. These observations imply that phytohormones derived from *R*. *pedestris* are involved in the green stem syndrome and transgenerational changes in *G*. *soja*. Further studies are needed to verify the involvement of these phytohormones and to clarify the mechanism and adaptive significance of the phenomenon both in plant and herbivore aspects.

## Supporting information

S1 Table Raw data of *Riptortus pedestris* captured by pheromone traps and pod maturation phenology of Glycine soja. (XLSX)

S2 Table Raw data of pod maturation time of Glycine soja infested with 0 (control), 6 (low density) or 12 (high density) *Riptortus pedestris* females. (XLSX)

S3 Table Raw data of pod and seed production in *Glycine soja* infested with 0 (control), 6 (low density) or 12 (high density) *Riptortus pedestris* females. (XLSX)

S4 Table Raw data of seed weight in *Glycine soja* infested with 0 (control), 6 (low density) or 12 (high density) *Riptortus pedestris* females. (XLSX)

S5 Table Raw data of pod maturation time of Glycine soja progeny of infested (high density) or non-infested (control) plants. (XLSX)

S6 Table Raw data of pod and seed production in the progeny of *Riptortus pedestris* infested (high density) and non-infested (control) *Glycine soja*. (XLSX)

S7 Table Raw data of seed weight in the progeny of *Riptortus pedestris* infested (high density) and non-infested (control) *Glycine soja*. (XLSX)
